# Post-traumatic soft tissue tumors: Case report and review of the literature a propos a Post-traumatic paraspinal desmoid tumor

**DOI:** 10.1186/1477-7819-6-28

**Published:** 2008-02-29

**Authors:** Sarit Cohen, Dean Ad-El, Ofer Benjaminov, Haim Gutman

**Affiliations:** 1 Department of Plastic Surgery, Rabin Medical Center, Beilinson Campus, Petah Tiqwa; and Sackler Faculty of Medicine, Tel Aviv University, Tel Aviv, Israel; 2 Department of Diagnostic Imaging, Rabin Medical Center, Beilinson Campus, Petah Tiqwa; and Sackler Faculty of Medicine, Tel Aviv University, Tel Aviv, Israel; 3 Department of Surgery, Rabin Medical Center, Beilinson Campus, Petah Tiqwa; and Sackler Faculty of Medicine, Tel Aviv University, Tel Aviv, Israel

## Abstract

**Background:**

Antecedent trauma has been implicated in the causation of soft tissue tumors. Several criteria have been established to define a cause-and-effect relationship. We postulate possible mechanisms in the genesis of soft tissue tumors following antecedent traumatic injury.

**Case presentation:**

We present a 27-year-old woman with a paraspinal desmoid tumor, diagnosed 3-years following a motor vehicle accident. Literature is reviewed.

**Conclusion:**

Soft tissue tumors arising at the site of previous trauma may be desmoids, pseudolipomas or rarely, other soft tissue growths. The cause-and-effect issue of desmoid or other soft tissue tumors goes beyond their diagnosis and treatment. Surgeons should be acquainted with this diagnostic entity as it may also involve questions of longer follow-up and compensation and disability privileges.

## Background

The etiology of most soft tissue tumors is unknown. Our search of the English literature revealed a few case reports of soft tissue tumors developing at the site of a previous traumatic injury [1-17]. Desmoid tumors, lipoma and lymphoma were among the tumors reportedly associated with such injuries.

We describe a young woman with a left paraspinal desmoid tumor at the site of a recent trauma, possibly associated with a cause-and-effect mechanism. We hope this study will shed more light on this phenomenon.

## Case presentation

**Figure 1:**
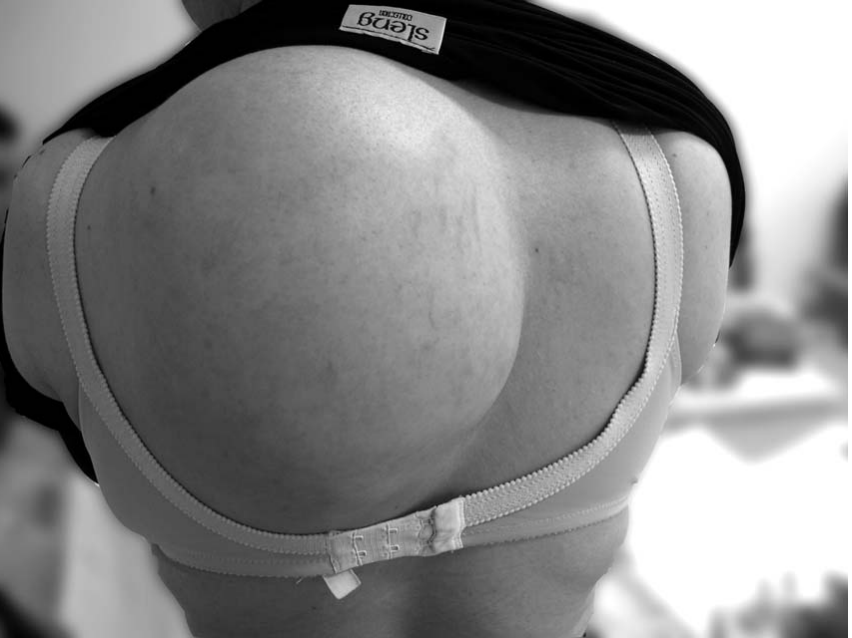
Large subcutaneous mass in the left paraspinal region.

A 27-year-old woman presented with a large subcutaneous mass in the upper back (Figure 1) of 8 months' duration.

Family history and past medical history were unremarkable. The patient reported that she had been involved in a motor vehicle accident 3 years previously, in which she sustained a brain concussion, fracture of the right lamina of the C-6 vertebra, and comminuted fractures of the left radius, ulna and femur.

**Figure 2:**
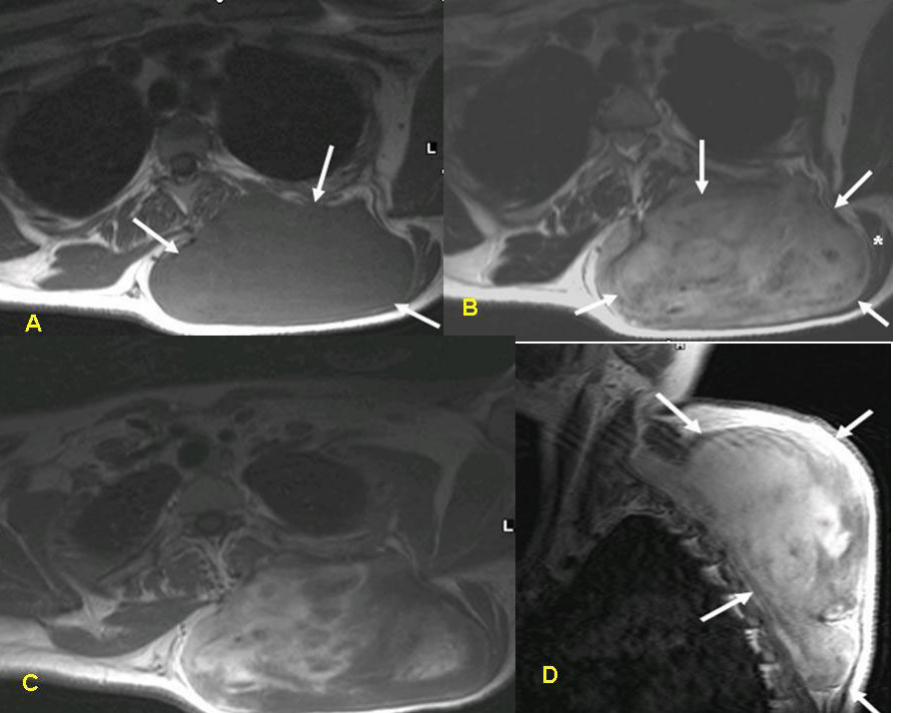
MRI of the tumor: T1W pre-(A) and post-(B) gadolinium injection, T2W (C) and T1W post gadolinium, sagittal view (D). The tumor (arrows) has a heterogenous appearance on T2W images and enhances with the injection of contrast material, demonstrating its vascularity. It is located beneath the trapezius muscle (asterisk) which is atrophic. The paraspinal muscle is compressed medially.

Physical examination revealed a firm mass measuring 15 × 10 cm, adherent to its surroundings, with no apparent pathological vasculature or satellite lesions. Cytological examination was inconclusive. Magnetic resonance imaging (MRI) demonstrated a solid space-occupying lesion measuring 12 × 4.8 × 7.6 cm, located in the left paraspinal region beneath the trapezium muscle (asterisk), compressing the paraspinal muscles medially (Figure 2). The tumor has a heterogeneous appearance on T_2 _weighted images and enhanced with the injection of contrast material, demonstrating its vascularity. Findings on core needle biopsy were compatible with desmoid tumor. Colonoscopy revealed no abnormalities.

Owing to the large size of the tumor and its close proximity to the spine, the initial treatment consisted of tamoxifen 20 mg twice daily and indomethacin 250 mg q8h. The treatment was well tolerated. However, after 4 months, neither subjective nor objective changes in tumor consistency or size were noted. The tamoxifen dosage was therefore doubled. Computerized tomography (CT) scan, 4 months later demonstrated tumor growth. There was no evidence of infiltration of adjacent bony structures or pulmonary metastases. The patient was offered surgery.

**Figure 3:**
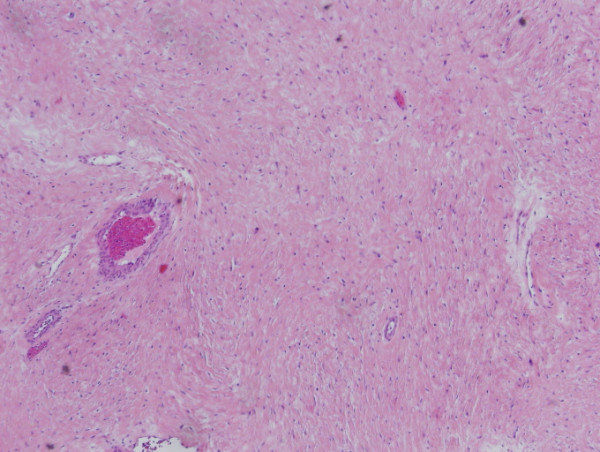
Histopathologic specimen demonstrating spindle cell proliferation without significant atypia or pleomorphism (HE × 40).

**Figure 4:**
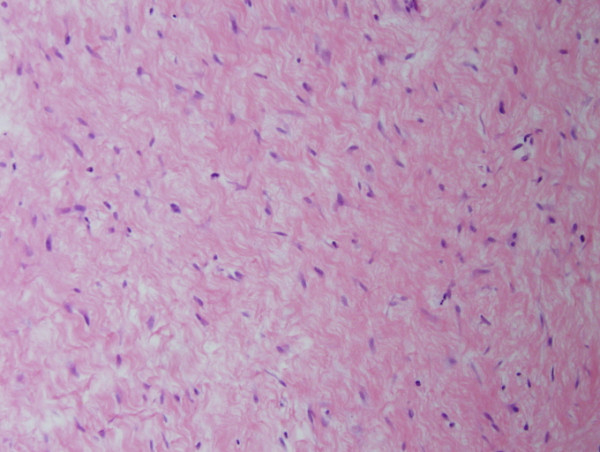
Photomicrograph at high power magnification (HE × 100).

The tumor was surgically excised. It measured 9 × 12 × 22 cm and weighed 1970 grams. It was relatively well circumscribed, with a fibrous consistency, and no areas of hemorrhage or necrosis. Microscopic study revealed relatively low (up to 2–3/10HPF) mitotic activity (Figure 3, 4). The surgical margins were clear. At present, 24 months postoperatively, the patient is tumor-free.

## Discussion

Desmoid tumor is a benign, locally aggressive neoplasm that arises from fascial or musculoaponeurotic tissue. It has a tendency to infiltrate surrounding tissue. The term 'desmoid', derived from the Greek "desmos" which means tendon-like was first employed by Müller [12] in 1838. Desmoid tumors account for 0.03% of all neoplasms [13,14], and 3.0% of all soft tissue tumors [15,16].

Patients with familial adenomatous polyposis (FAP) have a 1000-fold increased risk of developing desmoid tumors compared to the general population. The abdomen is the most common site of the tumors in this patient group, many times following a surgical insult.

The reported female: male ratio for sporadic desmoid tumors is 5:2 [17]; most women are affected during or after pregnancy. Reitamo *et al*., [13] found that 80% of desmoid tumors occur in females, 50% of them in the third to fifth decade of life. The female predominance is less prominent in patients with FAP [18,19].

Recently, It was found that virtually all desmoid tumors have somatic [beta]-catenin or adenomatous polyposis coli (APC) gene mutation leading to intranuclear accumulation of [beta]-catenin [20]. The expression of nuclear [beta]-catenin may play a role in the differential diagnosis of desmoid tumors from a host of fibroblastic and myofibroblastic lesions as well as from smooth muscle neoplasms [20]. The treatment of desmoid tumors is usually surgical. Local recurrences may occur even after clear margin resection. Distant metastases are extremely rare.

The pathogenesis of desmoid tumor may involve genetic abnormalities, sex hormones, and trauma [17], including surgical trauma, especially in patients with FAP [19]. One study found that 10–30% of all sporadic abdominal wall desmoid tumors occurred following surgical intervention. Half these tumors developed within 4 years of surgery [17].

Gebhart *et al*., [3] reported a case of desmoid tumor arising at the site of a total hip replacement. Desmoid tumors developing around silicone implants have also been described [13]. Skhiri *et al*., [1] reported a case of cervical desmoid following placement of an internal jugular catheter, and Wiel Marin *et al*., [2] described a thoracic desmoid tumor at the site of a previous rib fracture.

Traumatic injury has been implicated as a causative factor in the genesis of other soft tissues as well. Radhi *et al*., [6] reported 3 cases of diffuse centroblastic lymphoma at a site of previous surgery with metallic implants. Two of them were preceded by atypical lymphoid infiltrate.

In 1969, Brooke and MacGregor [21] suggested that lipoma may be secondary to trauma because of the prolapse of normal deep adipose tissue through a tear in the overlying Scarpa's fascia, namely, "pseudolipoma". Pseudolipoma consists of normal adipose tissue in an abnormal location, and is not considered a true lipoma because it is not encapsulated. Meggit and Wilson [22] reported 12 cases of post-traumatic so-called lipoma. They speculated that the tumors were the consequence of a rupture in the septa that normally surround adipose tissue. A later report by Herbert and DeGeus [23] described a young girl with an abdominal wall lipoma due to pressure from tightly fitting briefs. They demonstrated an anatomical defect in the Scarpa's fascia at the level of a perforating vessel with fat herniating through it.

The largest series of 24 pseudolipomas was reported by Rozner and Isaacs [24] in 1977, wherein scar contracture following a shearing fascial injury was the etiological mechanism. Penoff [25] described 3 cases of traumatic lipoma of the hip, although he found no anatomic confirmation of an injury to Scarpa's fascia.

In 1988, Dodenhoff [26] described a "saddle-bag deformity" of the right hip secondary to trauma. Post-traumatic lipoma was also reported by Elsahy [27] (5 cases) and David *et al*., [8] (10 cases). Signorini and Campiglio [9] described 9 cases of subcutaneous lipoma that appeared within a few months of a blunt trauma. They proposed that the differentiation of mesenchymal precursors (preadipocytes) to mature adipocytes – a process triggered by the trauma – could lead to the formation of subcutaneous lipoma.

Warren [28] listed several criteria defining a post-traumatic neoplasm: (a) prior integrity of the tumor site; (b) injury severe enough to initiate reparative proliferation of cells; (c) reasonable latent period; and (d) tumor compatible with the scar tissue and anatomic location of the injury. Ewing [29] suggested slightly different criteria to establish a cause/effect relationship: (a) authenticity and severity of the injury; (b) previous integrity of the wounded part; (c) tumor originating within the boundary of the injury; (d) histologic variety of tumor compatible with underlying scar tissue; and (e) proper latent period.

In our case, the wounded part (upper back) was previously tumor-free, the authenticity of the trauma was confirmed by MRI, the tumor originated within the boundary of the injury, and the latency period was reasonable. Furthermore, the desmoid histology was compatible with a scar or other reparative process. Thus, the tumor met the criteria of both Warren [28] and Ewing [29] for post-traumatic neoplasm.

## Conclusion

The cause-and-effect issue of desmoid or other soft tissue tumors goes beyond their diagnosis and treatment. It may also involve questions of longer follow-up and compensation and disability privileges.

Pseudolipomas are not real neoplasia, but they seem to account for the reports of the so-called post-traumatic lipomas. The post-injury local reparatory mechanisms better explain the creation of desmoid tumors, which, in these rare cases, seem to have lost control of cell growth, giving rise to a soft tissue tumor. The rarity of desmoid tumor, its specific biology, the well-documented association between abdominal wall desmoids and pregnancy, and even the tendency of surgery to induce new desmoid tumors in patients with FAP support the notion that trauma/tissue injury is a likely cause of at least, some of these tumors, including the one described here.

## Abbreviations

CT-computerized tomography; FAP-familial adenomatous polyposis; MRI-magnetic resonance imaging

## Competing interests

The author(s) declare that they have no competing interests.

## Authors' contributions

**CS **participated in drafting the manuscript, interpretation of data and conceptual design, **AD **conceived the study and participated in drafting the manuscript, **BO **carried out the imaging analysis and interpretation of data, **GH **carried out the surgical procedure, conceptual design, participated in drafting the manuscript and revised it critically for important intellectual content.

All authors read and approved the final manuscript.
